# Socioeconomic Status Transition Throughout Life and Risk of Dementia

**DOI:** 10.1001/jamanetworkopen.2024.12303

**Published:** 2024-05-21

**Authors:** Ryoto Sakaniwa, Kokoro Shirai, Dorina Cadar, Tami Saito, Katsunori Kondo, Ichiro Kawachi, Andrew Steptoe, Hiroyasu Iso

**Affiliations:** 1 Public Health, Department of Social Medicine, Osaka University Graduate School of Medicine, Osaka, Japan; 2 Department of Social and Behavioral Sciences, Harvard T. H. Chan School of Public Health, Boston, Massachusetts; 3 Department of Behavioural Science and Health, University College London, London, United Kingdom; 4 Centre for Dementia Studies, Department of Clinical Neuroscience, Brighton and Sussex Medical School, University of Sussex, Brighton, United Kingdom; 5 Center for Gerontology and Social Science, National Center for Geriatrics and Gerontology, Aichi, Japan; 6 Center for Preventive Medical Sciences, Chiba University, Chiba, Japan; 7 Institute for Global Health Policy Research, Bureau of International Health Cooperation, National Center for Global Health and Medicine, Tokyo, Japan

## Abstract

**Importance:**

Socioeconomic status (SES) is associated with dementia. However, the role of SES transitions in dementia is less explored; such evidence would be useful to understand whether social mobility is associated with healthy longevity at older ages.

**Objective:**

To investigate the association of lifetime SES transition with risk of dementia.

**Design, Setting, and Participants:**

This prospective cohort study, conducted from August 2010 to December 2016, used data from the Japan Gerontological Evaluation Study for participants aged 65 years or older from 31 different areas in Japan. Individuals with missing SES values, loss of follow-up, or new dementia onset 1 year or less from baseline were excluded. Data analysis was performed from April 2022 to April 2023.

**Exposure:**

Transitions in SES across the life course.

**Main Outcomes and Measures:**

The main outcome was risk of dementia incidence and corresponding loss or gain of dementia-free periods in a lifespan. The incidence of dementia was identified with a national registry of long-term nursing care services.

**Results:**

A total of 9186 participants (4703 men [51.2%]) were included. The mean (SD) age at baseline was 74.2 (6.0) years. Six SES transitions were identified: upward, stable-high, upper-middle, lower-middle, downward, and stable-low. During the follow-up period, 800 cases of dementia were identified. Many dementia risk factors, including lifestyle behaviors, comorbidities, and social factors, were associated with SES transition patterns. Compared with lower-middle SES, the lowest risk of dementia was observed for upward transition (hazard ratio [HR], 0.66; 95% CI, 0.57-0.74) followed by stable-high (HR, 0.77; 95% CI, 0.69-0.86), downward (HR, 1.15; 95% CI, 1.09-1.23), and stable-low (HR 1.45; 95% CI, 1.31-1.61) transition (*P* <  .001 for linearity); there was no association of upper-middle transition with risk of dementia (HR, 0.91; 95% CI, 0.79-1.03). The greatest increases in dementia-free years in the lifespan were also associated with upward SES transition (eg, 1.8 years [95% CI, 1.4-2.2 years] at age 65 years), while the downward transition was associated with the largest loss in lifetime dementia-free years at 75 years or older (eg, −1.4 years [95% CI, −2.4 to −0.4 years] at age 85 years).

**Conclusions and Relevance:**

This cohort study of Japanese older adults identified that upward and downward SES transitions were associated with risk of dementia and the length of dementia-free periods over the lifespan. The results may be useful to understand the association between social mobility and healthy longevity.

## Key Points

**Question:**

Are socioeconomic status transitions, particularly downward or upward transitions across the lifespan, associated with dementia?

**Findings:**

In this cohort study of 9186 participants, a downward socioeconomic status transition was associated with the highest loss of dementia-free years from the age of 75 years onward, and upward transitions were associated with the longest period of dementia-free years during the lifespan.

**Meaning:**

The findings suggest that both upward and downward socioeconomic status transitions are associated with dementia, which may be useful to understand the association between social mobility and healthy longevity.

## Teaser

This cohort study investigates whether transitions in socioeconomic status over the life course are associated with lifetime incidence of dementia and dementia-free years.

## Introduction

Dementia has emerged as a significant health burden affecting healthy longevity, particularly in industrialized countries.^1,2,3^ The influence of low socioeconomic status (SES) throughout life on the risk of dementia and the time to onset is a matter of concern. Various SES components, including lower childhood SES,^4^ limited educational attainment,^5^ a less demanding job,^6^ and lower household income in adulthood^7^ have been associated with a higher risk of dementia incidence. However, the changes across these SES components throughout the life course remain unclear; such evidence would be useful to understand the association between social mobility and healthy longevity.^8,9^ Prior studies have attempted to identify the association of overall life-course SES with dementia incidence^10,11,12,13,14^ using subjective classification^10,11,12^ or SES scores.^13,14^ However, there is a dearth of data-driven investigations on the impact of changing SES throughout the life course.

Therefore, we aimed to identify several independent SES transition subgroups within a nationwide cohort dataset to construct SES transition patterns over the life course through unsupervised clustering^15^ and investigated the association of SES classification over the life course with dementia incidence and dementia-free periods during the lifetime. This approach facilitated the data-driven classification of different measurement scales for the SES transition patterns.

## Methods

### Study Population

In this cohort study, we used data from the Japan Gerontological Evaluation Study (JAGES) cohort, which is extensively described elsewhere.^16,17^ In brief, the cohort recruited participants from 31 different areas in Japan from August 2010 to December 2016. Participants were aged 65 years or older, were not registered for long-term care or health and welfare social services, and lacked a dementia diagnosis. Informed consent was obtained from all participants, signified by completing and returning the self-administered questionnaire via mail. The Ethics Committee of the Research of Human Subjects at Nihon Fukushi University and Chiba University approved the study protocol. This study adhered to the Strengthening the Reporting of Observational Studies in Epidemiology (STROBE) reporting guideline for cohort studies.

### Measurements

All components of life-course SES and other variables at baseline were self-reported. Relative childhood SES was measured with the following question: “How do you rate your SES until the age of 15 years according to standards at that time?” Responses were arranged on a 3-point Likert scale, with 1 indicating “very-low/low”; 2, “middle”; and 3, “high/very-high.” We confirmed that these corresponding answers were associated with physical growth, such as adult height.^18^

Educational attainment was measured with the question, “How many years of formal education have you had?” The response options were less than 9, 10 to 12, or 13 or more years. Information on the longest-held job in middle age was ascertained by asking, “What was the job that you did for most of your working life?” Possible answers included household job; fisher, farmer, or other; technician, salesperson, or administrator; and expert or manager. The job category was classified based on the occupational classification provided by the Ministry of Internal Affairs and Communication, Japan. We confirmed a similar proportion of occupational classifications between our present study and the Japan census from 1985 to 1995.^19^

Participants were asked their pretax household income for the previous year, including a pension. We calculated an equivalized household income as the total household income divided by the square root of the number of household members. Each participant’s response was validated and assessed for correlation with the amount paid for social insurance in the past year (*P* < .001) because the Japanese health system adopts progressive taxation.^20^ We divided equivalized median household income into 4 quartiles as follows: $7217 (IQR, $5000-$8839) (quartile 1), $15 909 (IQR, $12 374-$19 445) (quartile 2), $24 748 (IQR, $20 207-$25 981) (quartile 3), and $38 690 (IQR, $31 754-$49 075) (quartile 4). The domestic Japanese currency was converted as 100 JPY = US $1.

We chose potential dementia risk factors. These included demographic variables (age at baseline and sex), physical characteristics and lifestyle behaviors (body mass index, sense of coherence, instrumental activities of daily living, sports participation, smoking status, and alcohol consumption behavior), history of comorbidities (hypertension, diabetes, dyslipidemia, osteoporosis, mental illness, cancer, cardiovascular disease, and stroke), and social factors (receiving social support, living alone, current marital status, number of close friends, and living area) at baseline.

### Dementia Diagnosis and Mortality

Dementia incidence and date of diagnosis were determined using the standardized assessment process under the registration for long-term care insurance throughout the follow-up period. This system is an official form of national social insurance for older individuals with disability in Japan. Candidates or their family can apply for registration irrespective of their current status, such as being already hospitalized, relocating to a nursing home, or living at their own home. Disability certification involves assessing the degree of functional disability using a questionnaire developed by the Japanese Ministry of Health, Labour and Welfare. The attending trained physician’s documentation on chronic medical conditions and daily-life physical and cognitive functions contributes to this assessment.^21,22,23^

Cognitive function measurements are standardized into 8 levels from lowest to highest: 0, I, IIa, IIb, IIIa, IIIb, IV, and M. These scores correlate with the Mini-Mental State Examination.^24^ A clinical dementia diagnosis was established when the disability level was IIa or higher (sensitivity, 83%; specificity, 92%). This was validated by veteran neuropsychiatrists through standardized clinical reviews of the International Psychogeriatric Association.^25^ Thus, dementia incidence and its date were defined when participants registered with a disability level of IIa or higher.

Information on all-cause mortality and date of death were obtained from death records from local municipalities throughout the study period. Participants were censored based on the date of death without a dementia diagnosis, the date of relocation to another municipality, or the date at the final follow-up on April 30, 2016.

### Statistical Analysis

To identify specific SES transition patterns over the life course and their distinct characteristics, we used unsupervised clustering analysis facilitating the data-driven classification of different measurement scales for the SES components.^15^ Differences in risk characteristics according to SES transition patterns were analyzed by 1-way analysis of variance and Kruskal-Wallis and χ^2^ tests. The hazard ratios (HRs) for dementia incidence and 95% CIs were examined by cumulative incidence function with competing risk analysis. Death without a dementia diagnosis was considered as a competing risk interest. For adjustment, we used inverse probability weights with multinominal propensity score analysis.^26^

To explore whether SES transitions were associated with the development of dementia risk factors, regression-based mediation analysis was used.^27^ Furthermore, we examined the potential loss or gain in the dementia-free period across the lifetime at the ages of 65, 75, 80, and 85 years for each SES transition. The losses or gains of dementia-free years, along with 95% CIs, were estimated using a multistate life table model and Monte Carlo simulation.^28,29^ Detailed information on statistical approaches and validation tests is given in the eMethods in Supplement 1. Statistical significance was set at *P* < .05 (2-tailed). Statistical analyses were performed from April 2022 to April 2023 using SAS, version 9.4 (SAS Institute Inc).

## Results

### Participant Characteristics

Among the 55 067 participants in the JAGES cohort, life-course SES assessment was conducted for a randomly selected subset of 12 398. We excluded 2959 individuals with missing SES values and 253 because of loss of follow-up or new dementia onset 1 year or less from baseline. Therefore, 9186 individuals were eligible for primary analyses (eFigure 1 in Supplement 1). During 6.4 years of follow-up (median, 5.8 years [IQR, 5.2-5.9 years]), a total of 800 cases of dementia developed. Of the 9186 individuals in the primary analysis, 688 (7.5%) died without a dementia diagnosis and 267 (2.9%) died with dementia. The mean age at baseline was 74.2 years (range, 65-102 years). A total of 4703 participants were men (51.2%) and 4483 were women (48.8%). Six classes were identified as the optimal number of life-course SES classifications: upward, stable-high, upper-middle, lower-middle, downward, and stable-low (eTable 1 in Supplement 1).

### Characteristics of Life-Course SES Transition Patterns

Table 1 shows the mean value and 95% CI of each SES category in accordance with the pattern of life-course SES transitions. Baseline characteristics of the participants according to SES transitions are summarized in Table 2. Participants with upward classifications tended to be men (567 of 867 [66.2%]), while a greater proportion of those with upper-middle (299 of 483 [61.9%]) and stable-low (861 of 1385 [62.2%]) classifications were women (*P* < .001). The difference in mean age among the 6 SES transitions was 3.3 years (from 72.9 to 76.2 years). Various dementia risk factors were associated with the life-course SES transitions.

**Table 1.** Mean SES Scores According to SES Transition Throughout the Life Course

SES measure	Score by pattern of SES transition, mean (95% CI) (N = 9186)						
	Upward	Stable-high	Upper-middle	Lower-middle	Downward	Stable-low
Total individuals, No. (%)	857 (9.3)	777 (8.5)	483 (5.3)	3608 (39.3)	2076 (22.6)	1385 (15.1)
Relative childhood SES^a^	1.0 (1.0-1.1)	2.5 (2.5-2.6)	1.7 (1.5-1.8)	1.3 (1.2-1.4)	2.9 (2.9-3.0)	1.7 (1.6-1.8)
Educational attainment^a^	1.9 (1.7-2.1)	2.4 (2.4-2.5)	2.4 (2.2-2.6)	2.0 (1.9-2.1)	2.1 (2.0-2.3)	1.1 (1.0-1.2)
Longest job type^a^	2.3 (2.1-2.5)	2.4 (2.2-2.6)	2.4 (2.1-2.7)	2.3 (2.1-2.5)	2.2 (1.9-2.5)	1.6 (1.5-1.7)
Equivalized household income at age ≥65 y^b^	4.0 (3.9-4.0)	4.0 (3.9-4.0)	2.9 (2.7-3.1)	2.6 (2.4-2.8)	1.7 (1.5-1.9)	1.3 (1.2-1.4)
Overall^c^	9.3 (8.7-9.8)	11.2 (11.0-11.5)	9.4 (8.5-10.2)	8.2 (7.6-8.8)	8.9 (8.3-9.6)	5.7 (5.3-6.2)

Abbreviation: SES, socioeconomic status.

^a^ On a 3-point scale.

^b^ On a 4-point scale.

^c^ Out of 13 possible points.

**Table 2.** Baseline Characteristics According to SES Transition Throughout the Life Course

Characteristic	Participants by pattern of SES transition (N = 9186)^a^						
	Upward	Stable-high	Upper-middle	Lower-middle	Downward	Stable-low	*P* value^b^
**Demographic characteristics**							
Total individuals, No. (%)	857 (9.3)	777 (8.5)	483 (5.3)	3608 (39.3)	2076 (22.6)	1385 (15.1)	<.001
Age at baseline, mean (SD), y	72.9 (5.2)	72.9 (5.9)	72.9 (5.7)	74.2 (5.7)	74.1 (5.6)	76.2 (6.2)	<.001
Sex							
Men	567 (66.2)	333 (42.9)	184 (38.1)	2060 (57.1)	1035 (49.9)	524 (37.8)	<.001
Women	290 (33.8)	444 (57.1)	299 (61.9)	1548 (42.9)	1041 (50.1)	861 (62.2)	
**Physical characteristics and lifestyle behaviors**							
Body mass index^c^							
<18.5	57 (6.7)	50 (6.4)	33 (6.9)	245 (6.8)	189 (9.1)	111 (8.0)	<.001
18.5-24.9	498 (58.1)	465 (59.9)	294 (60.9)	2103 (58.3)	1196 (57.6)	870 (62.8)	
25.0-29.9	285 (33.3)	249 (32.0)	148 (30.6)	1169 (32.4)	641 (30.9)	367 (26.5)	
≥30.0	16 (1.9)	13 (1.7)	8 (1.6)	90 (2.5)	50 (2.4)	37 (2.7)	
Sense of coherence score, mean (SD)^d^	24.4 (3.7)	23.4 (3.8)	22.9 (4.4)	21.7 (4.1)	22.9 (3.8)	21.2 (4.4)	<.001
Instrumental activities of daily living, median (IQR)^e^	12 (11-13)	13 (12-13)	13 (11-13)	12 (11-13)	12 (10-13)	11 (10-13)	<.001
Sports participation							
>1 Time/wk	170 (19.8)	164 (21.1)	91 (18.8)	451 (12.5)	390 (18.8)	141 (10.2)	<.001
1-2 Times/ mo	76 (8.9)	62 (8.0)	33 (6.8)	126 (3.5)	85 (4.1)	26 (1.9)	
≤1 Time/mo	535 (62.4)	466 (60.0)	271 (56.1)	2399 (66.5)	1304 (62.8)	1012 (73.1)	
Smoking status							
Current	95 (11.1)	71 (9.1)	44 (9.1)	473 (13.1)	214 (10.3)	166 (12.0)	<.001
Past	349 (40.7)	190 (24.5)	118 (24.4)	1155 (32.0)	797 (38.4)	143 (10.3)	
Never	354 (41.3)	457 (58.8)	273 (56.5)	1735 (48.1)	1131 (54.5)	812 (58.6)	
Alcohol consumption							
7 d/wk	207 (24.2)	130 (16.7)	76 (15.8)	761 (21.1)	407 (19.6)	188 (13.6)	<.001
3-6 d/wk	135 (15.8)	86 (11.1)	42 (8.7)	368 (10.2)	226 (10.9)	105 (7.6)	
<2 d/wk	110 (12.8)	71 (9.1)	51 (10.5)	426 (11.8)	241 (11.6)	120 (8.7)	
Never	374 (43.6)	451 (58.0)	308 (63.7)	1945 (53.9)	1123 (54.1)	911 (65.8)	
**Comorbidities**							
Hypertension	345 (40.3)	299 (38.5)	185 (38.3)	1519 (42.1)	822 (39.6)	575 (41.5)	.054
Diabetes	116 (13.5)	99 (12.8)	62 (12.9)	458 (12.7)	245 (11.8)	173 (12.5)	.057
Dyslipidemia	102 (11.9)	92 (11.8)	59 (12.3)	314 (8.7)	220 (10.6)	105 (7.6)	.002
Osteoporosis	58 (6.8)	44 (5.6)	24 (5.0)	249 (6.9)	168 (8.1)	141 (10.2)	<.001
Mental illness	4 (0.5)	6 (0.8)	5 (1.0)	36 (1.0)	27 (1.3)	18 (1.3)	<.001
Cancer	51 (6.0)	48 (6.2)	30 (6.3)	188 (5.2)	118 (5.7)	64 (4.6)	.02
Cardiovascular disease	123 (14.4)	116 (14.9)	79 (16.4)	570 (15.8)	309 (14.9)	220 (15.9)	.04
Stroke	12 (1.4)	13 (1.7)	9 (1.8)	65 (1.8)	33 (1.6)	25 (1.8)	.09
**Social factors**							
Received social supports	823 (96.0)	747 (96.2)	458 (94.9)	3410 (94.5)	1947 (93.8)	1259 (90.9)	<.001
Living alone	41 (4.8)	28 (3.6)	49 (10.1)	220 (6.1)	484 (23.3)	306 (22.1)	<.001
Current marital status							
Married	748 (87.3)	639 (82.3)	317 (65.7)	2746 (76.1)	1611 (77.6)	871 (62.9)	<.001
Bereaved	96 (11.2)	127 (16.4)	125 (25.8)	639 (17.7)	347 (16.7)	381 (27.5)	
Divorced	7 (0.8)	8 (1.0)	25 (5.2)	123 (3.4)	81 (3.9)	40 (2.9)	
Never married	5 (0.6)	1 (0.1)	16 (3.3)	97 (2.7)	60 (2.9)	65 (4.7)	
Close friends, median (IQR), No.	7 (4-10)	8 (4-10)	7 (4-10)	4 (1-10)	6 (4-10)	4 (4-7)	<.001
Living in urban area^f^	583 (68.0)	541 (69.6)	312 (64.6)	2049 (56.8)	1252 (60.3)	686 (49.5)	<.001

Abbreviation: SES, socioeconomic status.

^a^ Data are presented as number (percentage) of individuals unless otherwise indicated.

^b^ *P* values for differences were calculated with analysis of variance for variables with normal distribution, the Kruskal-Wallis test for variables with homogeneous distribution, and the χ^2^ test for variables with binominal distribution.

^c^ Calculated as weight in kilograms divided by height in meters squared. Less than 18.5 was considered underweight; 18.5 to 24.9, normal weight; 25.0 to 29.9, overweight; 30.0 or more, obese.

^d^ On a 30-point scale, with higher scores indicating greater coherence.

^e^ On a 13-point scale, with higher scores indicating greater independence.

^f^ Local population of 50 000 or more.

#### Upward Transition

Participants in the upward transition class (857 [9.3%]) experienced the lowest childhood SES and the second-lowest educational level but the highest equivalized household income later in life. A higher sense of coherence (mean [SD] score, 24.4 [3.7] of 30 points, with higher scores indicating greater coherence) and the lowest prevalence of mental illness (4 participants [0.5%]) were observed.

#### Stable-High Transition

Participants in the stable-high transition class (777 [8.5%]) had consistently high SES components from childhood to older age. These participants showed higher levels of healthy behaviors and more optimal social factors, and more lived in urban areas (541 [69.6%]).

#### Upper-Middle Transition

Participants in the upper-middle transition class (483 [5.3%]) had a stable upper-middle SES from childhood to older age. The majority were women (299 [61.9%]).

#### Lower-Middle Transition

Lower-middle was the most common SES transition pattern (3608 [39.3%]). The prevalence for each variable was neither the highest nor the lowest.

#### Downward Transition

Participants in the downward transition class (2076 [22.6%]) had high SES and educational levels in childhood but low household incomes later in life. A higher prevalence of underweight (189 participants [9.1%]), mental illness (27 [1.3%]), and living alone (484 [23.3%]) was observed compared with the other transition classes.

#### Stable-Low Transition

The stable-low transition class (1385 [15.1%]) was consistent with lower SES levels across the life course. Most participants in this class were women (861 [62.2%]). Lower levels of physical, behavioral, and social factors and more medication use for comorbidities were noted.

### Associations Between Life-Course SES and Dementia at Older Age

**Figure 1.**
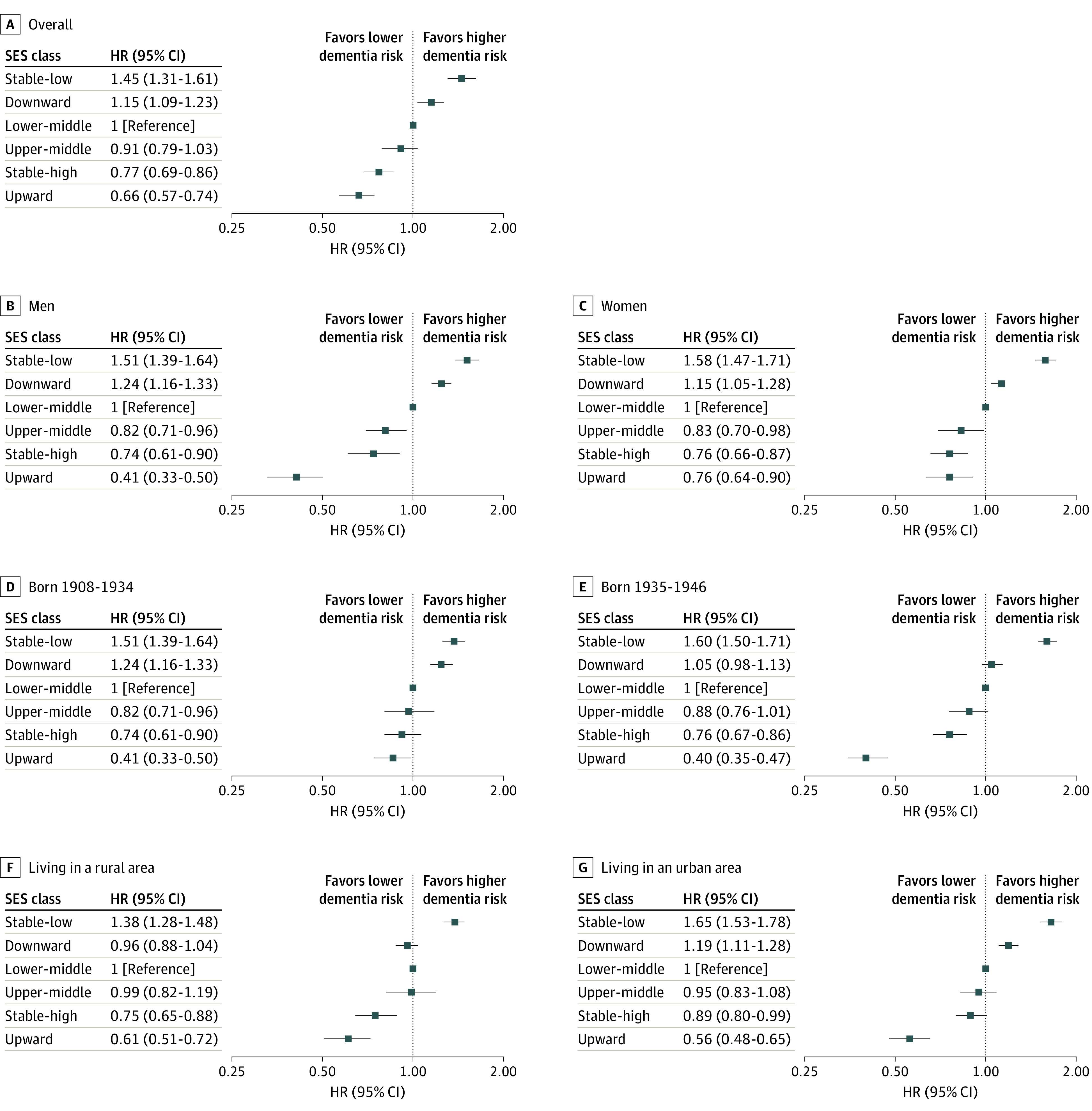
Hazard Ratios (HRs) for Dementia by Socioeconomic Status (SES) Transition Throughout the Life Course The HRs for dementia incidence were calculated with death among individuals without dementia as a significant competing risk factor (eTable 6 in Supplement 1). Age and sex HRs were controlled by multinominal inverse probability weighting for SES classification followed by age and sex. Rural was defined as a local population less than 50 000 and urban as 50 000 or more. For sex difference and difference in birth period, *P* < .001 for the interaction. For difference in living area, *P* = .02 for the interaction.

Figure 1 and eTable 2 in Supplement 1 show the associations between SES transitions and the incidence of dementia. We used the stable lower-middle SES pattern as a reference. The lowest risk of dementia incidence was observed in the upward SES model (HR, 0.66; 95% CI, 0.57-0.74), followed by stable-high (HR, 0.77; 95% CI, 0.69-0.86), downward (HR, 1.15; 95% CI, 1.09-1.23), and stable-low (HR, 1.45; 95% CI, 1.31-1.61) SES transitions (*P* < .001 for linearity); there was no association of upper-middle SES transition with dementia risk (HR, 0.91; 95% CI, 0.79-1.03). The associations remained for upward, stable-high, downward, and stable-low SES after adjusting for age and sex: HRs of 0.66 (95% CI, 0.57-0.74) for upward, 0.77 (95% CI, 0.69-0.86) for stable-high, 1.15 (95% CI, 1.09-1.23) for downward, and 1.45 (95% CI, 1.31-1.61) for stable-low; there was no association of upper-middle transition with risk of dementia (HR, 0.91; 95% CI, 0.79-1.03). The SES transitions were associated with greater risk of dementia incidence in men than in women (*P* < .001 for interaction), in individuals born later than in those born earlier (*P* < .001 for interaction), and in participants living in urban areas than in those living in rural areas (*P* = .02 for interaction).

The study found that SES transitions mediated the development of other potential risk factors for dementia. All of the physical characteristics and lifestyle behaviors, comorbidities, and social factors were mediators of associations between dementia risk and upward (mediation effect size: physical and lifestyle characteristics, −16.5% [95% CI, −22.4% to −6.2%]; comorbidities, −10.6% [95% CI, −25.6% to −8.7%]; and social factors, −13.6% [95% CI, −22.4% to 6.2%]), stable-high (physical and lifestyle characteristics, −12.6% [95% CI, −21.4 to −4.0]; comorbidities, −10.9% [95% CI, −8.5% to 14.7%]; and social factors, −8.6% [95% CI, −4.0% to 14.0%]), and stable-low (physical and lifestyle characteristics, 11.8% [95% CI, 8.1%-14.6%]; comorbidities, 12.8% [95% CI, 8.6%-15.3%]; and social factors, 11.1% [95% CI, 8.1%-14.6%]) SES transitions. Only social factors were major mediators of the association of dementia risk with downward SES transition (13.8%; 95% CI, 7.6%-21.6%) (Table 3).

**Table 3.** Mediation Analysis for SES Transition and Other Potential Dementia Risk Factors

Dementia risk factor	Mediation effect size by pattern of SES transition, % (95% CI)^a^					
	Upward	Stable-high	Upper-middle	Lower-middle	Downward	Stable-low
Overall	−40.8 (−66.7 to −18.6)	−32.1 (−46.6 to −19.5)	−17.7 (−57.1 to 15.4)	Reference	32.3 (14.1 to 53.6)	35.7 (26.4 to 46.2)
Physical characteristics and lifestyle behaviors^b^	−16.5 (−22.4 to −6.2)	−12.6 (−21.4 to 4.0)	−5.3 (−12.6 to 8.2)	Reference	4.4 (−12.6 to 8.2)	11.8 (8.1 to 14.6)
Comorbidities^c^	−10.6 (−25.6 to −8.7)	−10.9 (−8.5 to 14.7)	−11.2 (−19.3 to 6.5)	Reference	14.1 (−21.1 to 1.2)	12.8 (8.6 to 15.3)
Social factors^d^	−13.6 (−22.4 to 6.2)	−8.6 (−4.0 to 14.0)	−1.2 (−12.6 to 8.2)	Reference	13.8 (7.6 to 21.6)	11.1 (8.1 to 14.6)

Abbreviation: SES, socioeconomic status.

^a^ Adjusted for age and sex and further for other potential mediators; 95% CIs were estimated with bootstrapping analysis.

^b^ Body mass index, sense of coherence, instrumental activities of daily living, sports participation, smoking status, and alcohol consumption behavior.

^c^ Hypertension, diabetes, dyslipidemia, osteoporosis, mental illness, cancer, cardiovascular disease, and stroke.

^d^ Receiving social support, living alone, current marital status, number of close friends, and living area.

**Figure 2.**
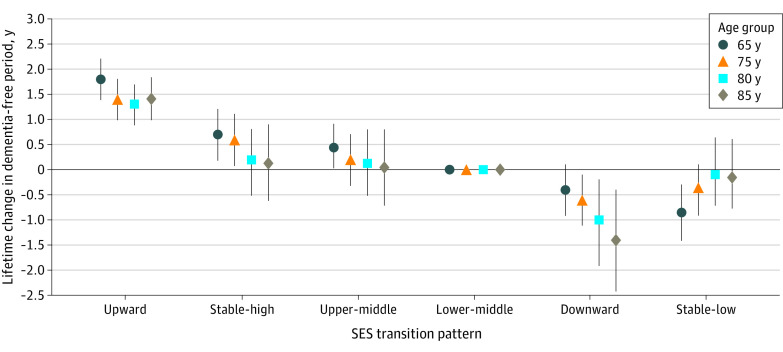
Loss or Gain of Dementia-Free Periods Over the Lifetime and Socioeconomic Status (SES) Transitions Throughout the Life Course Results are adjusted by sex. Whiskers indicate 95% CIs, estimated with Monte Carlo simulation.

We estimated the losses or gains in the dementia-free period over the participants’ lifetimes due to the pattern of SES transitions; the largest gain in dementia-free lifetime years was observed for upward SES transition across all age categories. The gain of dementia-free years in the upward transition class was 1.8 (95% CI, 1.4-2.2) at age 65 years, 1.4 (95% CI, 1.0-1.8) at 75 years, 1.3 (95% CI, 0.9-1.7) at 80 years, and 1.4 (95% CI, 1.0-1.8) at 85 years. Benefits and disadvantages of the SES transitions were slightly attenuated in older ages. However, for the downward SES transition, the largest loss of dementia-free years was at 75 years or older. For example, the change in the dementia-free lifetime period at age 75 years was −0.6 years (95% CI, −1.1 to −0.1 years) but was −1.4 years (95% CI, −2.4 to −0.4 years) at the age of 85 years (Figure 2 and eFigure 2 in Supplement 1).

### Additional Analyses and Validation

In the single-isolate analysis, educational attainment was inversely associated with the risk of dementia, while other components showed no association (eTable 3 in Supplement 1). When addressing potential selection bias, baseline characteristics and dementia rates were similar between the included (n = 9186) and excluded (n = 44 556) individuals (eTable 4 in Supplement 1). In the assessment of the association of time with dementia onset, a hazard proportionality was found (eFigure 3 in Supplement 1), and the primary results remained unchanged even when excluding individuals who were censored in the first 2 to 5 years (eTable 5 in Supplement 1). When we investigated survivor bias at entry, we observed no association between SES classifications and the risk of nondementia mortality except for the upward transition (eTable 6 in Supplement 1). Random split sampling validation affirmed the robustness of clustering features. Considering unmeasured confounders, minimum HRs were generally greater than primary results, especially for the upward and stable-high classes; the E-values^30^ were 3.18 and 2.40, respectively (eFigure 4 in Supplement 1).

## Discussion

Our analysis included 9186 older individuals aged 65 years or older; the lowest risk of dementia and greatest gain of dementia-free periods were observed in participants with an upward SES trend. In contrast, those who underwent downward SES transitions exhibited the largest reduction in healthy lifespan periods at an older age.

Previous studies using subjective classifications to explore the life course of SES consistently found that maintaining a low SES throughout life was linked to the highest risk of dementia.^10,11,12^ The impact of upward or downward SES transitions over the life course has not been well examined, although some studies do exist. For instance, in the Sacramento Area Latino Study on Aging cohort of 1789 older US participants from Mexico, in which childhood SES, educational attainment, and occupational status were considered, no differences in the risk of dementia between upward SES (HR, 1.17; 95% CI, 0.56-2.43) and downward SES (HR, 0.80; 95% CI, 0.54-1.18) compared with stable-low SES were observed.^10^ Additionally, the Uppsala Birth Cohort Multigenerational Study observed varied dementia risks associated with the combination of high educational level and low occupational status (HR, 0.82; 95% CI, 0.68-0.98) but not with the combination of low educational level and high occupational status (HR, 0.88; 95% CI, 0.64-1.21).^11^

The strength in clustering analyses in a life-course study lies in its ability to identify data-driven subgroups with optimal prevalence and group numbers, which can detect more anomaly-based classifications compared with subjective classification.^15^ Importantly, this statistical approach allows the identification of unremarkable and/or minor classes but shows unique characteristics. The lowest dementia incidence in our study was observed in participants with upward SES transitions, with a focal point in the gain of lifetime periods without dementia diagnosis. This report marks the first documentation, to our knowledge, of the benefits of upward SES patterns during the life course for dementia prevention. For instance, successful experiences throughout the life course may delay the acceleration of cognitive aging by enhancing stress resistance.^31^ Notably, our study showed that (1) individuals with upward SES transition had the highest sense of coherence and the lowest prevalence of mental disorders and (2) physical and lifestyle behaviors largely functioned as indirect effects. Both our results and previous results^31^ provide evidence for social mobility in relation to healthy longevity at an older age.

In our study, we found that mediation effect sizes for physical characteristics and lifestyle behaviors, comorbidities, and social factors varied among the SES transitions. Physical characteristics and lifestyle behaviors were major mediating factors in upward, stable-high, and stable-low SES transitions, while only social factors were mediating factors in downward SES transitions.

The SES transitions were associated with the greatest risk of dementia among those born later, men, or urban residents compared with those born earlier, women, or rural residents. Birth cohort and sex differences in a social context for dementia have been studied in Western countries, but research in other regions remains limited.^13,32,33^ For example, the English Longitudinal Study of Aging showed that the association of socioeconomic inequalities with dementia was more prominent in English individuals born between 1926 and 1946 than in those born between 1900 and 1925.^13^ In contrast to our results, higher social deprivation at older ages was associated with a 30% increased risk of dementia in women but not in men, according to a 3-city cohort in France.^32^ Similarly, educational attainment in early life was associated with a lower risk of dementia among women according to a meta-analysis of 11 prospective cohort studies in the UK.^33^ Although similar clustering or transition analyses were not conducted in these studies, our study provided new evidence for regional differences in the association of life-course SES transition with the risk of dementia between Asian and Western populations.

### Strengths and Limitations

The main strengths of our study lay in its novel analysis of SES transition and dementia, supported by higher response rates at primary screening, well-standardized dementia detection, and a large cohort of older participants, and that it was conducted as a prospective cohort study in multiregional areas in Japan. Furthermore, Japan is the country that shows the highest life expectancy in the world, with higher social mobility^2^ and a long-term commitment in a single organization (Statistics Bureau of Japan),^19^ which may provide the scientific evidence required for other countries and in future studies.

The following limitations should also be noted. First, the JAGES data are from an observational cohort based on a self-reported questionnaire. Although we conducted several validation tests, the possibility of misclassification and unmeasured bias should be taken into account. Second, the regional value of equivalized household income and specific job categorization in Japanese society might not be applicable to other societies. However, the ranking SES value within a country would be valid for the analysis. Third, we did not have information on dementia subtypes. Fourth, survivor selection bias upon entry is a common issue in gerontologic studies,^34^ which may require careful interpretation. Fifth, we excluded 3212 (25.9%) of 12 398 participants due to incomplete SES assessment or the occurrence of dementia within 1 year or less of follow-up. This exclusion may have led to selection bias, although several sensitivity analyses consistently supported the primary results. Sixth, our follow-up length was short; therefore, the results may have been susceptible to reverse causation. Seventh, the inherent limitation of unsupervised clustering analysis lies in labeling groups based on subjective interpretation.

## Conclusions

In this cohort study of Japanese adults aged 65 years or older, we identified distinct SES transition patterns throughout life and found that upward transitions were associated with lower risk of dementia and increases in length of dementia-free periods over the life course, whereas the reverse was true for downward transitions. To our knowledge, this is the first report highlighting changes in SES classes from childhood and their specific associations with a dementia diagnosis at an older age.

## Notes

Supplement 1. **eTable 1.** Definition of the Number of Groups **eTable 2.** HRs of Dementia Incidence According to SES Transition Throughout the Life Course **eTable 3.** HRs of Dementia Incidence and Each SES Component **eTable 4.** Comparison of Baseline Characteristics and Incident Rate of Dementia Between Included and Excluded Participants **eTable 5.** HRs of Dementia According to SES Transition Throughout the Life Course, Excluding Early Absence of Follow-Up **eTable 6.** HRs of Non-Dementia Mortality According to SES Transition Throughout the Life Course **eFigure 1.** Flowchart for Current Analysis **eFigure 2.** Sex-Specific Loss or Gain of Dementia-Free Periods Over Lifetime and SES Transition Throughout the Life Course **eFigure 3**. Cumulative Incidence Function for Dementia Incidence or Nondementia Death and SES Transition Throughout the Life Course **eFigure 4.** E-Values of Primary Analysis According to the Pattern of SES Transition **eMethods.** Detailed Information for Statistical Approaches
eReferences

Supplement 2.
Data Sharing Statement
