# Incorrect Spelling of Author Surname

**DOI:** 10.1001/jamanetworkopen.2024.24508

**Published:** 2024-06-27

In the Original Investigation titled “Socioeconomic Status Transition Throughout Life and Risk of Dementia,”^1^ published May 21, 2024, in the byline, author affiliations, and conflict of interest disclosures, the surname of Dorina Cadar, PhD, was incorrectly given as Cador. This article has been corrected.
