# Supplementary material for: Socioeconomic Status Transition Throughout Life and Risk of Dementia
Source: JAMA Netw Open. 2024 May 21;7(5):e2412303. doi: 10.1001/jamanetworkopen.2024.12303 (PMC11109776; doi:10.1001/jamanetworkopen.2024.12303)
Supplement: Supplement 1. — eTable 1. Definition of the Number of Groups eTable 2. HRs of Dementia Incidence According to SES Transition Throughout the Life Course eTable 3. HRs of Dementia Incidence and Each SES Component eTable 4. Comparison of Baseline Characteristics and Incident Rate of Dementia Between Included and Excluded Participants eTable 5. HRs of Dementia According to SES Transition Throughout the Life Course, Excluding Early Absence of Follow-Up eTable 6. HRs of Non-Dementia Mortality According to SES Transition Throughout the Life Course eFigure 1. Flowchart for Current Analysis eFigure 2. Sex-Specific Loss or Gain of Dementia-Free Periods Over Lifetime and SES Transition Throughout the Life Course eFigure 3. Cumulative Incidence Function for Dementia Incidence or Nondementia Death and SES Transition Throughout the Life Course eFigure 4. E-Values of Primary Analysis According to the Pattern of SES Transition eMethods. Detailed Information for Statistical Approaches eReferences [file jamanetwopen-e2412303-s001.pdf]

Supplementary Online Content

Sakaniwa R, Shirai K, Cador D, et al. Socioeconomic status transition throughout life and risk of dementia. *JAMA Netw Open*. 2024;7(5):e2412303. doi:10.1001/jamanetworkopen.2024.12303

eTable 1. Definition of the Number of Groups
eTable 2. HRs of Dementia Incidence According to SES Transition Throughout the Life Course
eTable 3. HRs of Dementia Incidence and Each SES Component
eTable 4. Comparison of Baseline Characteristics and Incident Rate of Dementia Between Included and Excluded Participants
eTable 5. HRs of Dementia According to SES Transition Throughout the Life Course, Excluding Early Absence of Follow-Up
eTable 6. HRs of Non-Dementia Mortality According to SES Transition Throughout the Life Course
eFigure 1. Flow Chart for Current Analysis
eFigure 2. Sex-Specific Loss or Gain of Dementia-Free Periods Over Lifetime and SES Transition Throughout the Life Course
eFigure 3. Cumulative Incidence Function for Dementia Incidence or Nondementia Death and SES Transition Throughout the Life Course
eFigure 4. E-Values of Primary Analysis According to the Pattern of SES Transition
eMethods. Detailed Information for Statistical Approaches
eReferences

This supplementary material has been provided by the authors to give readers additional information about their work.

© 2024 Sakaniwa R et al. *JAMA Network Open*.

eTable 1. The definition of the number of groups.

No. of group (s)	Spectrum for maximum log-likelihood	Minimum proportion of group (s), %
1	N.A.	100
2	0.454	44.1
3	1.352	20.4
4	1.298	24.0
5	1.376	14.1
6	1.431	5.3
7	1.046	7.9
8	1.24	1.3
9	1.123	1.2
10	0.961	5.1
11	0.944	4.2
12	0.993	1.3
13	Not converged	N.A.
14	N.A.	N.A.
15	N.A.	N.A.

N.A.: not assessed.

© 2024 Sakaniwa R et al. *JAMA Network Open*.

eTable 2. HRs of dementia incidence according to SES transition throughout the life course [a,b].

Pattern of SES transition	Upward	Stable-high	Upper-middle	Lower-middle	Downward	Stable-low	*P* linearity [c]
Overall (n=9 186)							
Person-years	4 708	4 235	2 562	19 742	2 561	7 301	
No. at risk	857	777	483	3 608	2 076	1 385	
No. of cases	39	54	39	288	162	218	
Incidence rate, %	4.6	6.9	8.1	8.0	7.8	15.7	<0.001
Age-sex HR (95% CI)	0.66 (0.57-0.74)	0.77 (0.69-0.86)	0.91 (0.79-1.03)	Reference	1.15 (1.09-1.23)	1.45 (1.31-1.61)	<0.001
Men (n=4 705) [d]							
Incidence rate, %	4.0	6.9	10.3	8.1	8.8	14.0	<0.001
Age HR (95% CI)	0.41 (0.33-0.50)	0.74 (0.61-0.90)	0.82 (0.71-0.96)	Reference	1.24 (1.16-1.33)	1.51 (1.39-1.64)	<0.001
Women (n=4 481) [d]							
Incidence rate, %	5.5	7.0	6.7	7.8	6.8	16.8	<0.001
Age HR (95% CI)	0.76 (0.64-0.90)	0.76 (0.66-0.87)	0.83 (0.70-0.98)	Reference	1.15 (1.05-1.28)	1.58 (1.47-1.71)	<0.001
Born 1908-1934 (n=3 406) [d]							
Incidence rate, %	12.4	14.8	14.6	15.0	16.1	22.%	<0.001
Age-sex HR (95% CI)	0.86 (0.75-0.98)	0.92 (0.81-1.06)	0.97 (0.81-1.17)	Reference	1.24 (1.15-1.35)	1.37 (1.26-1.48)	<0.001
Born 1935-1946 (n=5 780) [d]							
Incidence rate, %	1.3	2.8	3.2	4.2	3.6	8.7	<0.001
Age-sex HR (95% CI)	0.40 (0.35-0.47)	0.76 (0.67-0.86)	0.88 (0.76-1.01)	Reference	1.05 (0.98-1.13)	1.60 (1.50-1.71)	<0.001
Living in rural area (n=3 786) [d,e]							
Incidence rate, %	4.4	7.2	7.4	9.3	6.9	16.3	<0.001
Age-sex HR (95% CI)	0.61 (0.51-0.72)	0.75 (0.65-0.88)	0.99 (0.82-1.19)	Reference	0.96 (0.88-1.04)	1.38 (1.28-1.48)	<0.001
Living in urban area (n=5 787) [d]							
Incidence rate, %	4.6	6.8	9.0	7.0	8.4	15.2	<0.001
Age-sex HR (95% CI)	0.56 (0.48-0.65)	0.89 (0.80-0.99)	0.95 (0.83-1.08)	Reference	1.19 (1.11-1.28)	1.65 (1.53-1.78)	<0.001

SES: socioeconomic status, HR: hazard ratio, 95% CI: 95% confidence interval.

[a] Age-sex HRs (95% CIs) are controlled by multinominal inversed probability for SES classification weighting, followed by age and sex.

[b] The HRs of dementia incidence are considered to impact non-dementia death as a significant competing risk in eTable 6.

[c] P linearities are estimated with the Cochran–Armitage test for incidence rate.

[d] $P_{interaction}$ for sex difference and difference in birth period, <0.001 each; $P_{interaction}$ for difference in living area=0.020.

[e] A rural area is defined as a rural population <50 000, while an urban area as ≥50 000 of local population.

© 2024 Sakaniwa R et al. *JAMA Network Open*.

eTable 3. HRs of dementia incidence and each SES component.[a]

Subjects with single-isolated SES	Age and sex-adjusted HR (95% CI)	HR (95% CI) adjusted further for each SES component
Relative childhood SES (per one category)	0.99 (0.92-1.06)	0.98 (0.92-1.08)
Educational attainment (per one category)	0.83 (0.76-0.91)	0.86 (0.79-0.98)
Longest job types (per one category)	0.92 (0.83-1.02)	0.94 (0.84-1.08)
Equivalized household income in current (per one categories)	0.93 (0.88-0.99)	0.98 (0.91-1.05)

SES: socioeconomic status, HR: hazard ratio, 95% CI: 95% confidence interval

[a] The HRs of dementia incidence are considered to impact non-dementia death as a significant competing risk.

© 2024 Sakaniwa R et al. *JAMA Network Open*.

eTable 4. Comparison of baseline characteristics and incident rate of dementia between included and excluded participants. [a]

**Participants**	Included	Excluded [c]
**Demography**		
No. at risk, n	9 186	44 556
Age at baseline, mean (SD) years	73.3 (5.4)	74.1 (5.1)
Males, n (%)	4 703 (51.2)	20 006 (44.9)
**Incidence rates**		
No. of incidence	800	4 696
Person-years	49 832	237 966
1 000 Incidence/person-years	16.1	19.7
**Physical and behavioral characteristics** [b]		
Body mass index, mean (SD) kg/m^2	22.9 (3.2)	22.8 (3.3)
Underweight, n (%)	615 (6.7)	3 297 (7.4)
Normal, n (%)	5 493 (59.8)	26 021 (58.4)
Overweight, n (%)	2 856 (31.1)	14 258 (32.0)
Obesity, n (%)	220 (2.4)	980 (2.2)
Sense of coherence, mean (SD) /34points	28.3 (4.1)	22.3 (4.2)
Instrumental activity of daily living, median (IQR) /17 points	16 (14-17)	16 (14-17)
Sports participation >1 times/week, n (%)	1 414 (15.4)	6 148 (13.8)
Ever smokers, n (%)	3 995 (43.5)	17 466 (39.2)
Daily alcohol intake, n (%)	1 598 (17.4)	8 554 (19.2)
**Comorbidities**		
Hypertension, n (%)	3 729 (40.6)	18 045 (40.5)
Diabetes mellitus, n (%)	1 184 (12.9)	5 614 (12.6)
Dyslipidemia, n (%)	1 001 (10.9)	4 411 (9.9)
Osteoporosis, n (%)	551 (6.0)	3 252 (7.3)
Mental illness, n (%)	955 (10.4)	5 302 (11.9)
Cancer, n (%)	523 (5.7)	2 406 (5.3)
Cardiovascular disease, n (%)	1 423 (15.5)	6 950 (15.6)
Stroke, n (%)	183 (2.0)	757 (1.7)
**Social factors**		
Receives social support, n (%)	8 644 (94.1)	41 883 (94.0)
Living alone, n (%)	1 028 (11.2)	5 614 (12.6)
Marital status, %	6 889 (75.0)	31 011 (69.6)
No. of close friends, median (IQR) n	7 (4-10)	7 (4-10)
Living urban (local population ≥50 000), n (%)	5 419 (59.0)	26 912 (60.4)

SD: standard deviation, IQR: interquartile range

[a] The variables with normal distribution are presented as means (standard deviation), homogeneity distribution as median (interquartile ranges [IQR]), and binominal distribution as no .at risk (percentages).

[b] Underweight, normal, overweight, and obesity are defined according to the body mass index <18.5, 18.5-24.9, 25.0-29.9 and ≥30.0 kg/m^2, respectively.

© 2024 Sakaniwa R et al. *JAMA Network Open*.

[c] The excluded population is defined as those with incomplete or no life-course socioeconomic status information and those who can attend follow-up for ≥1 years.

© 2024 Sakaniwa R et al. *JAMA Network Open*.

eTable 5. HRs of dementia according to SES transition throughout the life course, excluding early absence of follow-up.[a,b]

Pattern of SES transition	Upward	Stable-high	Upper-middle	Lower-middle	Downward	Stable-low
Excluded ≤ 2 years of follow-up						
No. at risk	837	761	467	3 510	2 013	1 328
No. of dementia	28	49	30	249	131	182
Age-sex HR (95% CI)	0.43 (0.38-0.49)	0.77 (0.70-0.85)	0.88 (0.78-0.99)	Reference	1.01 (0.95-1.07)	1.52 (1.44-1.61)
Excluded ≤ 3 years of follow-up						
No. at risk	818	735	454	33 384	2 956	2 346
No. of dementia	22	37	25	205	96	239
Age-sex HR (95% CI)	0.40 (0.34-0.47)	0.80 (0.71-0.90)	1.09 (0.95-1.25)	Reference	1.09 (1.02-1.17)	1.67 (1.55-1.80)
Excluded ≤ 4 years of follow-up						
No. at risk	796	705	436	3 258	1 879	1 168
No. of dementia	13	23	11	148	65	84
Age-sex HR (95% CI)	0.39 (0.33-0.47)	0.83 (0.72-0.95)	0.94 (0.78-1.12)	Reference	1.20 (1.10-1.29)	1.41 (1.28-1.55)
Excluded ≤ 5 years of follow-up						
No. at risk	775	681	415	3 129	1 802	1 093
No. of dementia	5	12	3	84	26	35
Age-sex HR (95% CI)	0.48 (0.37-0.61)	0.88 (0.73-1.07)	0.61 (0.44-0.84)	Reference	1.40 (1.25-1.56)	1.49 (1.31-1.70)

SES: socioeconomic status, HR: hazard ratio, 95% CI: 95% confidence interval.

[a] Age-sex HRs (95% confidence intervals [CIs]) are controlled by multinominal inversed probability for SES classification weighting, followed by age and sex.

[b] The HRs of dementia incidence is considered to impact non-dementia death as a significant competing risk.

© 2024 Sakaniwa R et al. *JAMA Network Open*.

eTable 6. HRs of non-dementia mortality according to SES transition throughout the life course. [a,b]

Pattern of SES transition	Upward	Stable-high	Upper-middle	Lower-middle	Downward	Stable-low	P linearity [c]
Overall (n=9 186)							
Person-years	4 708	4 235	2 562	19 742	2 561	7 301	
No. at risk	857	777	483	3 608	2 076	1 385	
No. of death	49	55	36	291	140	117	
Mortality rate, %	5.7	7.1	7.5	8.1	7.8	8.5	0.081
Age-sex HR (95% CI)	0.72 (0.54-0.98)	1.02 (0.76-1.45)	1.03 (0.73-1.46)	Reference	0.90 (0.74-1.11)	1.07 (0.86-1.33)	0.221
Males (n=4 705) [d]							
Mortality rate, %	7.4	11.4	11.4	10.8	9.1	13.4	0.046
Age HR (95% CI)	0.75 (0.53-1.04)	1.19 (0.79-1.58)	1.02 (0.65-1.60)	Reference	0.85 (0.67-1.09)	1.09 (0.84-1.44)	0.460
Females (n=4 481) [d]							
Mortality rate, %	2.4	3.8	5.0	4.5	4.4	5.5	0.054
Age HR (95% CI)	0.61 (0.28-1.32)	0.84 (0.50-1.43)	1.05 (0.61-1.82)	Reference	1.02 (0.71-1.48)	1.02 (0.70-1.50)	0.256
Born 1908-1934 (n=3 406) [d]							
Mortality rate, %	10.8	8.9	12.2	13.1	8.9	10.3	0.560
Age-sex HR (95% CI)	0.84 (0.56-1.26)	0.77 (0.50-1.20)	1.14 (0.75-1.72)	Reference	0.72 (0.54-1.00)	0.90 (0.68-1.19)	0.791
Born 1935-1946 (n=5 780) [d]							
Mortality rate, %	3.6	6.1	4.0	5.3	5.7	6.5	0.052
Age-sex HR (95% CI)	0.66 (0.42-1.04)	1.33 (0.90-1.98)	0.91 (0.49-1.69)	Reference	1.13 (0.85-1.50)	1.34 (0.95-1.90)	0.054
Living in rural area (n=3 786) [d,e]							
Mortality rate, %	4.7	6.3	6.4	8.5	6.4	7.9	0.227
Age-sex HR (95% CI)	0.61 (0.33-1.09)	0.87 (0.51-1.49)	0.85 (0.47-1.56)	Reference	0.82 (0.60-1.13)	0.90 (0.65-1.25)	0.496
Living in urban area (n=5 787) [d]							
Mortality rate, %	6.2	7.4	8.0	7.7	6.9	9.1	0.037
Age-sex HR (95% CI)	0.79 (0.55-1.13)	1.08 (0.76-1.53)	1.19 (0.77-1.81)	Reference	0.96 (0.74-1.25)	1.20 (0.89-1.62)	0.233

SES: socioeconomic status, HR: hazard ratio, 95% CI: 95% confidence interval.

[a] Age-sex HRs (95% CIs) are controlled by multinominal inversed probability for SES classification weighting, followed by age and sex.

[b] The HRs of non-dementia morality is considered to impact dementia incidence as a significant competing risk in Figure 1 & eTable 2.

[c] P linearities are estimated with the Cochran–Armitage test for mortality rate.

[d] $P_{interaction}$ for sex difference, difference in birth period, and difference in living area are 0.521, 0.382, and 0.714, respectability.

[e] A rural area is defined as a rural population <50 000, while an urban area as ≥50 000 of local population

© 2024 Sakaniwa R et al. *JAMA Network Open*.

eFigure 1. Flow chart for current analysis.

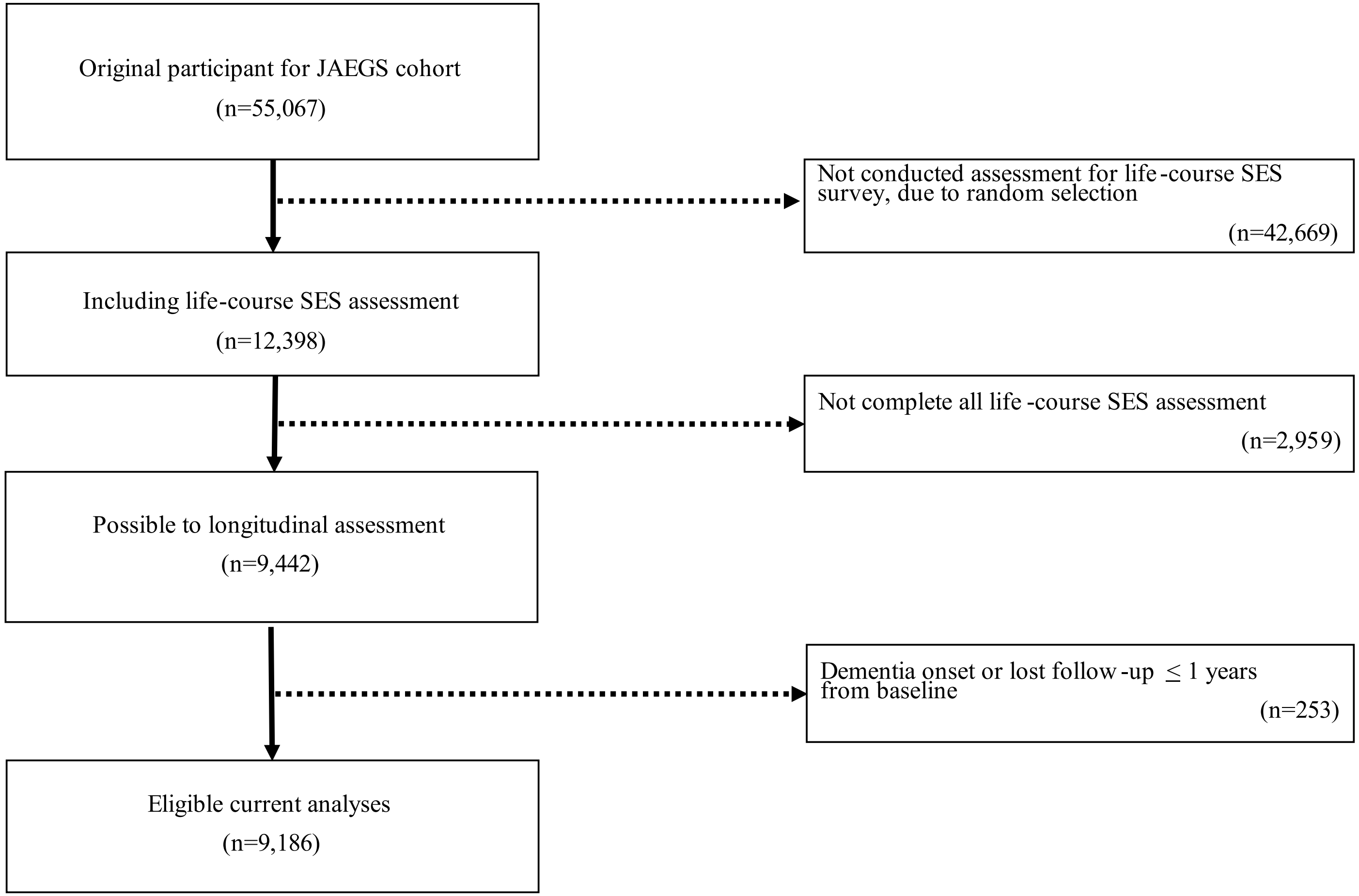

JAGES: Japan Gerontological Evaluation Study, SES: socioeconomic status

[a] Inclusion criteria for principal recruitment is: 1) No-diagnosis for dementia and functional disability and 2) Aged 65 years or more at baseline.

© 2024 Sakaniwa R et al. *JAMA Network Open*.

eFigure 2. Sex-specific loss or gain of dementia-free periods over lifetime and SES transition throughout life course. [a]

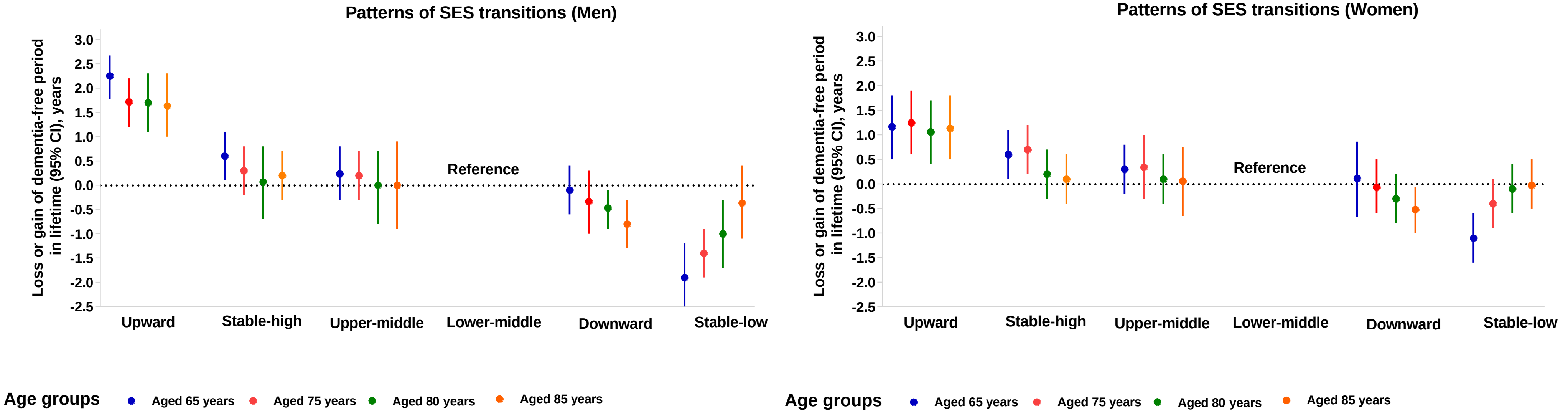

SES: socioeconomic status, 95% CI: 95% confidential interval.

[a] 95% CIs are examined with Monte–Carlo simulation.

© 2024 Sakaniwa R et al. *JAMA Network Open*.

eFigure 3. Cumulative incidence function for dementia incidence or non-dementia death and SES transition throughout the life course.[a,b]

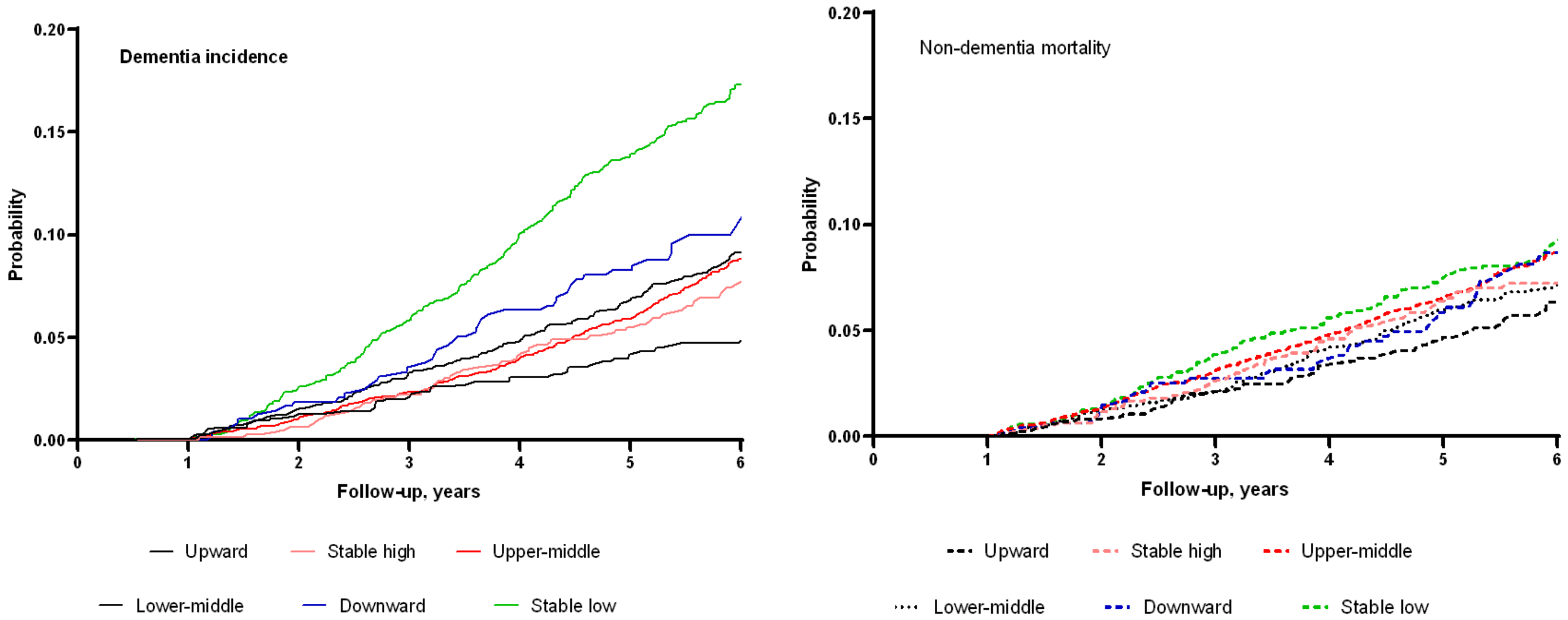

[a] *P* for difference <0.001 for dementia incidence and 0.102 for non-dementia morality.

[b] *P* for difference is examined with Gray's test

© 2024 Sakaniwa R et al. *JAMA Network Open*.

eFigure 4. E-values of primary analysis, according to the pattern of SES transition. [a]

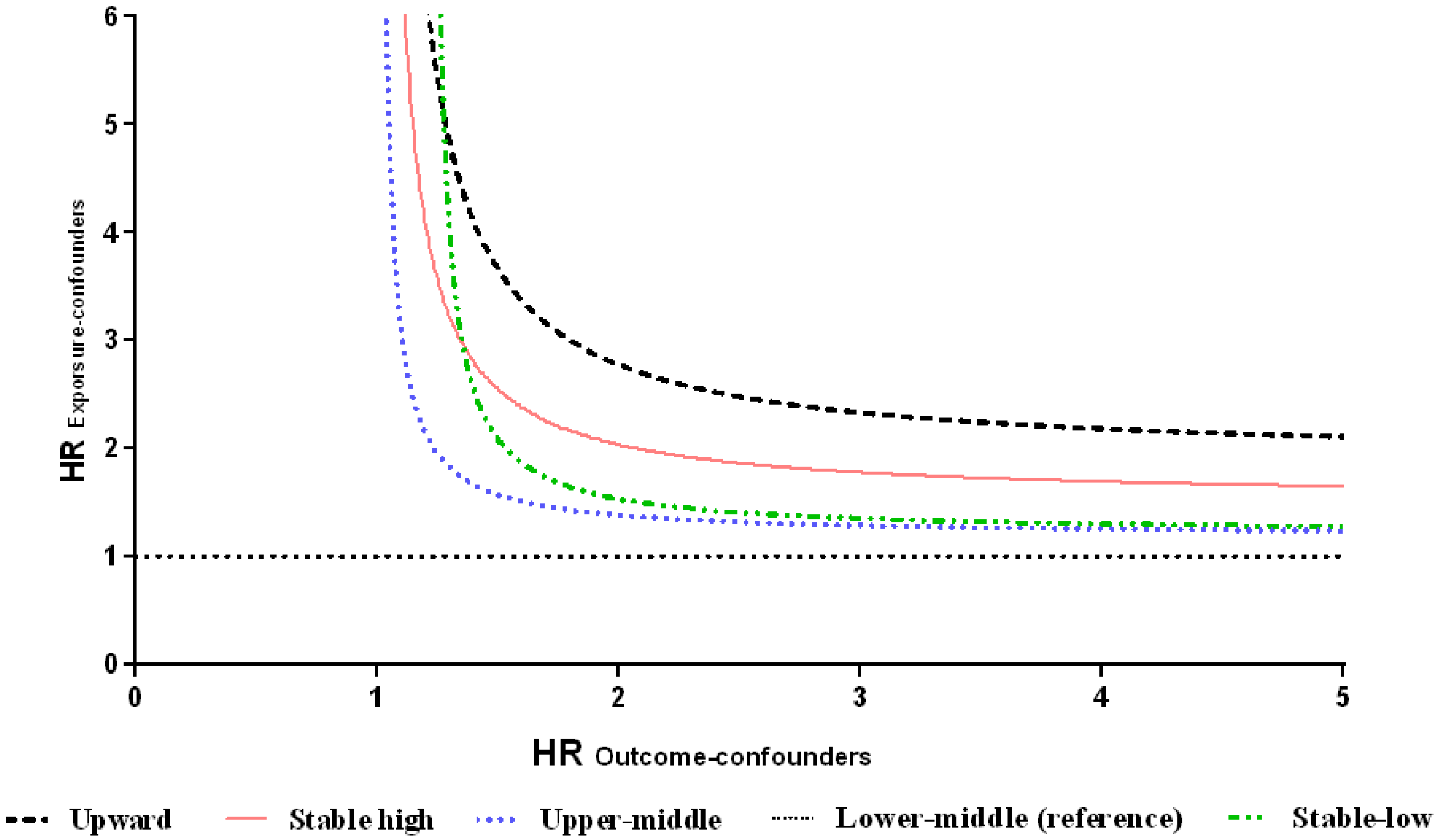

SES transition	Upward	Stable-high	Upper-middle	Lower-middle	Downward	Stable-low
E-value, HR (lower or upper limit of 95% CI)	3.18 (2.45)	2.40 (1.96)	Not assessed	Reference	1.57 (1.37)	2.52 (2.13)

[a] E-values are defined as the joint minimum required for HR and 95% CI that unmeasured confounders need to move the multivariable HR for the pattern of SES transition in the life course and dementia incidence to the null in Figure 1.

SES: socioeconomic status, HR: hazard ratio, 95% CI: 95% confidence interval, N.A: Not assessed.

© 2024 Sakaniwa R et al. *JAMA Network Open*.

eMethods. Detail Information for Statistical Approaches.

Unsupervised Clustering Analysis

To identify the distinct number of socioeconomic status (SES) transition patterns over the life course, we employed unsupervised clustering analysis, allowing data-driven classification across different measurement scales and multiple vector components.[1] In the current analysis, we defined four mode-vectors: relative childhood SES, educational attainment, longest job types, and current equivalized household income.

The optimal number of groups was determined using the spectrum for maximum likelihood. The unsupervised clustering analysis was iteratively performed, starting with one group, and progressing until the spectrum for the number of groups no longer converged or until it reached a number of 15 groups. Oversample regulation was implemented, ensuring a minimum group size of ≥5.0%. Therefore, the optimal number of SES transition groups was defined by satisfying the following criteria: 1) the highest spectrum of maximum likelihood before converged or among 15 candidates and 2) the minimum proportion of group size >5.0%.

Individual classification was based on the distance to the nearest neighbors, confirmed by posterior probabilities for the current categorized groups of all participants. We identified six classifications under the two conditions mentioned above. The labelling of groups was based on careful interpretation by the characteristics of the four SES components, resulting in labels such as "upward," "stable-high," "upper-middle," "lower-middle," "downward," and "stable-low." Posterior probabilities ranged from 0.82 to 0.99. Sensitivity analyses confirmed the robustness of classification through 25% random sampling using unsupervised clustering analyses repeated 200 times. Additionally, we assessed the impact of excluded subjects with incomplete SES assessments by including them in the analysis while maintaining a fixed number of six classifications. For investigations into relative risk, mediation analysis, and the loss or gain of dementia-free periods, we used the lower-middle group as a reference, given its majority classification (39.3% overall).

Characteristics of Dementia Risk Factors, According to SES Transition

Differences in dementia risk characteristics according to life-course SES transitions were assessed with the mean (standard deviation) for normally distributed variables, median (interquartile ranges) for homogenous variables, and number at risk (percentages) for binominal variables. We tested these p-values for differences by one-way analysis of variance and the Kruskal–Wallis and chi-square tests, respectively.

The listed dementia risk characteristics and their displayed format were demographic information (age at baseline [continuous] and sex [male or female]), and potential mediators included 1) physical characteristics and lifestyle behavior (body mass index [<18.5, 18.5-24.9, 25.0-29.9, or >30.0 kg/m^2], sense of coherence [continuous], instrumental activities of daily living [contentious], sports participation [>1 times/week, 1-2 times/week, or <1 time/month and no participation], smoking status [never, past, or current], and alcohol consumption behavior [7 days/week, 3-6 days/week, and less than 2 days/week or never drinking]), 2) history of comorbidities (hypertension [yes or no], diabetes mellitus [yes or no], dyslipidemia [yes or no], osteoporosis [yes or no], mental illness [yes or no], cancer [yes or no], cardiovascular disease [yes or no], and stroke [yes or no]), and 3) social factors (received social support [yes or no], living alone [yes or no], current marital status [married, bereavement, divorce, or never married], number of close friends [continuous], and living area [urban: local population ≥50 000 or rural: <50 000]) at baseline.

The Risk of Dementia Assessment

To assess the risk of dementia based on patterns of SES transition over the life course, we initially calculated the absolute incidence rate and examined its linearity using the Cochran–Armitage test. SES transition patterns were ordered according to the

© 2024 Sakaniwa R et al. *JAMA Network Open*.

results from the absolute incidence rate. Subsequently, hazard ratios (HRs) and 95% confidence intervals (CIs) were determined through cumulative incidence function analysis with competing risk analysis, considering non-dementia death as a competing risk of interest. Cumulative incidence curves for dementia incidence and non-dementia death are presented in eFigure 3. Hazards proportionality and linearity assumptions of HR were confirmed through Gray's test and through the log-log survival curves and the fractional polynomials test, respectively.

For age and sex adjustment, considering significant differences in age and sex within the six classifications, and the challenge of control through conventional multivariable adjustment models, we employed inverse probability weight analysis. Individual propensity scores for each of the six SES classifications were derived through multinomial logistic regression with age and sex adjustment, enabling the use of propensity score analysis when comparative groups exceed three.[2] We truncated outliers on both sides of probability weights close to the mean probability weight of 1.00. We fixed probability weights higher than the 99th percentile at the 99th percentile value and those lower than the 1st percentile at the 1st percentile value.[3] This approach aimed to control the generation of narrower CIs with improved coverage.[4] To account for historical differences in social context, sex, and current regional disparities, we conducted interaction analyses between SES transition and the risk of dementia incidence, considering birth difference periods (born between 1908-1935 or 1935-1945), sex difference (male or female), and living area difference (urban or rural area) models. For sensitivity analysis, we performed 200-times split-sample validations to validate the risk of dementia incidence and the pattern of life-course SES transition (training-to-test ratio: 1:4). To address survival bias at entry, we investigated the risk of non-dementia mortality according to SES transitions. Additionally, reversal causation was explored by excluding individuals who were censored in the first 2 to 5 years.

Mediation Analysis

For mediation analysis, we utilized regression-based mediation analysis, enabling consideration of multivariable adjustment, such as age and sex, and further for potential dementia risk factors, respectively.[5,6] The indirect effect of potential dementia risk factors was assessed using multivariable adjusted coefficients of expose-mediators (e.g., *a*) × mediators-outcome (e.g., *a'*), respectively. The direct effect was examined with multivariable adjusted coefficient of exposure-outcome (e.g. *d'*). For example, the total indirect effect of physical characteristics and lifestyle behaviors was the sum of indirect effect (body mass index, sense of coherence, instrumental activities of daily living, sports participation, smoking status, and alcohol consumption behavior]. The total effect defined as the sum of the multivariable adjusted indirect and direct effects (e.g., *aa'+bb'+cc'+…+d'*). Thus, mediation effects of physical characteristics and lifestyle behaviors were calculated as indirect effect/total effect (e.g., *aa'+bb'+cc'/aa'+bb'+cc'+…+d'*). The use of mediation factors and their categorization shown in baseline dementia risk characteristics in Table 2.

In this analysis, all contentious variables were standardized within a range between 0.0 and 1.0. Further, since we used two different regression models, 1) logistic regression for binominal outcomes (e.g., dementia incidence, history of hypertension, and social support) and linear regression for contentious outcome (e.g., sense of confidence, instrumental activities of daily living, and number of close friends), we converted these different coefficients, using a formula from the SAS official website.[7] Furthermore, the 95% CI was estimated with bootstrapping analysis by 10 000 runs.[5]

Investigation of Gain or Lost Dementia-free Periods Over Lifespan

To investigate the loss or gain in dementia-free periods over the lifetime at the ages of 65, 75, 80, and 85 years for each SES transition, we created period multistate life tables to estimate the differences in lifespan and years lived free of dementia or with dementia diagnosis by the presence of SES transitions. The multistate life table includes the information from different ages and birth cohorts, incorporating the three states: "dementia-free," "with dementia diagnosis," and "death." The possible transitions

© 2024 Sakaniwa R et al. *JAMA Network Open*.

categories from age [X] to [X+1] were: 1) from dementia-free to dementia diagnosis, 2) from dementia-free to death, and 3) from with dementia diagnosis to death.[8] Due to the small number of participants aged 100 years or more, this multistate lifetable was closed and concluded at age of 100+ years.

To evaluate the differences in the risk of dementia incidence and mortality among participants aged 65, 75, 80, and 85 years according to SES transitions, we calculated the overall and age-specific transition probabilities for each transition from age [X] to [X+1] with a Bayesian model. Next, we examined the sex-adjusted survival curves according to each SES transition using the "Gompertz" distribution model. Here, we used separate lifespan Cox proportional hazards models to assess the risk of mortality without dementia diagnosis, risk of dementia incidence, and risk of mortality among patients with dementia diagnosis. Finally, we estimated combined HRs with the overall transition probability curves according to SES transitions, to calculate total lifespan and lifespan with or without dementia.

The number of lost or gained dementia-free life years at ages of 65, 75, 80, and 85 years according to SES transitions were estimated based on differences in the area under the curve, which represented the lost or gain dementia-free life years between age [X] and 100 years of age.[9] Further, we calculated the CIs for all loss of dementia-free life years and their differences in lifespan with or without dementia diagnosis, using Monte Carlo simulation, bootstrapping with 200 runs.[10]

These combined statistical approaches allowed us to estimate and compare specific disease-free periods in the participants' total lifespans, according to different conditional groups. Similar calculations to our analyses were described elsewhere.[10,11] In this approach, we extended the follow-up period to between the date at dementia diagnosis and the date at death during the follow-up period for participants who were diagnosed with dementia.

Other Validation Analysis

In addressing missing variables, we employed multiple imputation through a chained equations model. To assess selection and survival bias, we compared baseline characteristics and dementia incidence rates between the analyzed cohort of 45 625 individuals and those excluded from the analysis due to a lack of SES information and follow-up <1 year. Additionally, we investigated the single-isolated association between each SES component and dementia incidence.

© 2024 Sakaniwa R et al. *JAMA Network Open*.

eReferences

1. Syarif I, Prugel-Bennett A, Wills G. Unsupervised clustering approach for network anomaly detection. In: Benlamri R, ed. *Communications in Computer and Information Science*. Berlin, Heidelberg: Springer; 2012:135-145. doi:10.1007/978-3-642-30507-8_13
2. Mccaffrey DF, Griffin BA, Almirall D, Slaughter ME, Ramchand R, Burgette LF. A Tutorial on propensity score estimation for multiple treatments using generalized boosted models. *Stat Med*. 2013;32(19):3388-3414.
3. Cole SR, Hernán MA. Constructing inverse probability weights for marginal structural models. *Am J Epidemiol*. 2008;168(6):656-664.
4. Robins JM, Hernán MÁ, Brumback B. Marginal structural models and causal inference in epidemiology. *Epidemiology*. 2000;11(5):550-560.
5. Hayes AF, Preacher KJ. Statistical mediation analysis with a multicategorical independent variable. *Br J Math Stat Psychol*. 2014;67(3):451-470.
6. Lee H, Cashin AG, Lamb SE, et al. A guideline for reporting mediation analyses of randomized trials and observational studies: The AGReMA Statement. *JAMA*. 2021;326(11):1045-1056.
7. SAS SUPPORT Mediation analysis. https://support.sas.com/kb/59/081.html. Accessed February 6, 2024.
8. Peeters A, Mamun AA, Willekens F, Bonneux L. A cardiovascular life history. A life course analysis of the original Framingham Heart Study cohort. *Eur Heart J*. 2002;23(6):458-466.
9. Library WO, Andersen PK. Decomposition of number of life years lost according to causes of death. *Stat Med*. 2013;32(30):5278-5285.
10. Rapsomaniki E, Timmis A, George J, et al. Blood pressure and incidence of twelve cardiovascular diseases: lifetime risks, healthy life-years lost, and age-specific associations in 1·25 million people. *Lancet*. 2014;383(9932):1899-1911.
11. Dhana K, Nano J, Ligthart S, et al. Obesity and Life expectancy with and without diabetes in adults aged 55 years and older in the Netherlands: A prospective cohort study. *PLoS Med*. 2016;13(7): e1002086. doi: 10.1371/journal.pmed.1002086

© 2024 Sakaniwa R et al. *JAMA Network Open*.
