# Supplementary material for: Socioeconomic Status Transition Throughout Life and Risk of Dementia
Source: JAMA Netw Open. 2024 May 21;7(5):e2412303. doi: 10.1001/jamanetworkopen.2024.12303 (PMC11109776; doi:10.1001/jamanetworkopen.2024.12303)
Supplement: Supplement 2. — Data Sharing Statement [file jamanetwopen-e2412303-s002.pdf]

Data Sharing Statement

Sakaniwa. Socioeconomic Status Transition Throughout Life and Risk of Dementia. *JAMA Netw Open*. Published May 21, 2024. doi:10.1001/jamanetworkopen.2024.12303

Data
Data available: No

Additional Information
Explanation for why data not available: The data sharing will be made for bona fide researchers who have appropriate request. Those wishing to request the access should contact to Prof. Hiroyasu Iso, of the corresponding author in present study.
